# In Vitro Bioactivity and Biocompatibility of Bio-Inspired Ti-6Al-4V Alloy Surfaces Modified by Combined Laser Micro/Nano Structuring

**DOI:** 10.3390/molecules25071494

**Published:** 2020-03-25

**Authors:** Chen Li, Yong Yang, Lijun Yang, Zhen Shi, Pengfei Yang, Guanghua Cheng

**Affiliations:** 1 College of Mechanical and Electrical Engineering, Shaanxi University of Science and Technology, Xi’an 710021, China; yanglijun@sust.edu.cn (L.Y.); 20150312105@sust.edu.cn (Z.S.); 2 State Key Laboratory of Transient Optics and Photonics, Xi’an Institute of Optics and Precision Mechanics, CAS, Xi’an 710119, China; yangyong@opt.ac.cn; 3 Key Laboratory of Space Radiobiology of Gansu Province, Key Laboratory of Heavy Ion Radiation Biology and Medicine of Chinese Academy of Sciences, Institute of Modern Physics, CAS, Lanzhou 730000, China; yangpengfei@impcas.ac.cn; 4 School of Electronics and Information, Northwestern Polytechnical University, Xi’an 710072, China; guanghuacheng@nwpu.edu.cn

**Keywords:** bioinspired surfaces, bioactivity, biocompatibility, femtosecond laser, LIPSS, cell alignment, cell adherence, Ti-6Al-4V

## Abstract

The bioactivity and biocompatibility play key roles in the success of dental and orthopaedic implants. Although most commercial implant systems use various surface microstructures, the ideal multi-scale topographies capable of controlling osteointegration have not yielded conclusive results. Inspired by both the isotropic adhesion of the skin structures in tree frog toe pads and the anisotropic adhesion of the corrugated ridges on the scales of Morpho butterfly wings, composite micro/nano-structures, including the array of micro-hexagons and oriented nano-ripples on titanium alloy implants, were respectively fabricated by microsecond laser direct writing and femtosecond laser-induced periodic surface structures, to improve cell adherence, alignment and proliferation on implants. The main differences in both the bioactivity in simulated body fluid and the biocompatibility in osteoblastic cell MC3T3 proliferation were measured and analyzed among Ti-6Al-4V samples with smooth surface, micro-hexagons and composite micro/nano-structures, respectively. Of note, bioinspired micro/nano-structures displayed the best bioactivity and biocompatibility after in vitro experiments, and meanwhile, the nano-ripples were able to induce cellular alignment within the micro-hexagons. The reasons for these differences were found in the topographical cues. An innovative functionalization strategy of controlling the osteointegration on titanium alloy implants is proposed using the composite micro/nano-structures, which is meaningful in various regenerative medicine applications and implant fields.

## 1. Introduction

Advancements of surface treatments in metallic biomaterials have allowed the production of better or longer lasting dental and orthopedic implants. Titanium alloy Ti-6Al-4V is the popular material for dental and orthopedic implants due to its good biocompatibility, excellent corrosion resistance, high load-bearing capacity and lightness [1,2]. However, Ti-6Al-4V is a bioinert biomaterial and its osseointegration ability is not good enough, which may result in implant failure over the long term. To enhance the bioactivity and biocompatibility of titanium alloy implants, surface modification strategies have attempted to mimic the characteristics of bone and trigger specific cellular responses to induce cell adherence, alignment and proliferation [3,4,5].

Various methods have been used to improve the osseointegration capability of the titanium alloy implants by enhancing bioactivity and biocompatibility on their surfaces, such as powder mesh metallurgical sintering [6], plasma spray processing [7], surface blasting [8], reactive ion etching (RIE) [9], electrochemical micromachining (EMM) [10], laser surface structuring [11] and chemistry surface treatment [12]. Different from all the aforementioned chemical-based techniques with the chemical contamination and limited topography, laser surface structuring is suitable for the selective modification of surfaces and allows the rapid generation of a specific 3-D structure at the micrometer and nanometer level with a low risk of contamination [13,14]. Among various laser sources, long-pulse fiber lasers including the millisecond and microsecond lasers are cheap and widely used in the industry, and are suitable for large-area microscale surface structures with a complicated shape [15]. In recent years, femtosecond (fs) lasers have become an advanced tool for metallic material processing with higher precision, a reduced heat-affected zone and a smaller amount of debris [16,17]. An amazing property of fs lasers is the ability to trigger the formation of self-organized quasi-periodic surface patterns, generally called laser-induced periodic surface structures (LIPSS) [18]. Fs-LIPSS provide a fast and precise tool for nanoscale surface structuring with anisotropic properties, such as directional wettability [13]. Therefore, we adopted a hybrid method combining the expensive fs laser nano-structuring with limited efficiency and the low-cost microsecond laser micro-structuring with high efficiency to generate Ti-6Al-4V implant surfaces with desired bioactivity and biocompatibility.

**Figure 1:**
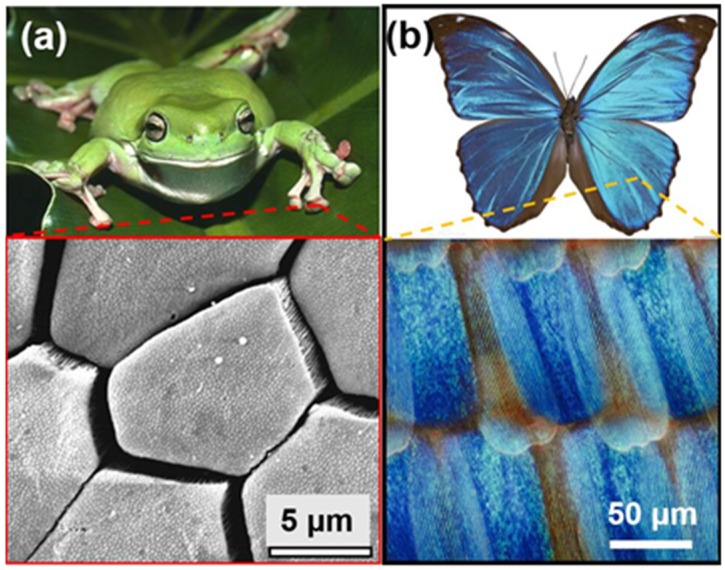
(**a**) Photograph of tree frog, with an inset showing the SEM of hexagonal epithelial cells with much finer pegs on toe pad epidermis [24]; (**b**) Photograph of Morpho butterfly, with an inset showing the scales with corrugated ridges of its wings.

Most commercial implant systems use micro-rough surface patterns due to the advantage of rough surfaces in stimulating osseointegration [19,20]. However, although the cellular responses to implant surfaces are being progressively elucidated, the ideal multi-scale topographies capable to control osseointegration have not yielded conclusive results [21,22,23]. Nature offers a variety of micro/nano-scale hierarchical structures with remarkable efficiency for tailored functionalities that could be used in promoting cell adhesion and proliferation, such as lotus leaf structure [22,23]. However, different from super-hydrophobic lotus leaf structures, there are other natural hierarchical structures for better controlling adhesion, such as the skin structures of tree frog toe pads [24,25] and the scale structure of Morpho butterfly wings [26,27] in Figure 1. For example, attachment forces on smooth wet leaves are significantly enhanced by tree frog toe pads (Figure 1a), which are characterized by peg-studded hexagonal cells separated by deep channels, and by much finer pegs on the flattened surface of each hexagonal cell [24]. Moreover, the wings of Morpho butterflies show the directional adhesion for water droplets, which is related to the wing’s scales with corrugated microscale ridges as shown in Figure 1b [27]. Inspired by the remarkable adhesion of both the skin structures in tree frog toe pads and the corrugated ridges on the scales of Morpho butterfly wings, biomimetic hierarchical surface structures on titanium alloy implants are fabricated by combined laser surface structuring in this work, to improve cell adherence, orientation and proliferation on implants. At first, a microsecond laser was used to generate the primary array of micro-hexagons like hexagonal cells of tree frog toe pads, and secondly, a femtosecond laser was then applied to directly induce nano-ripples with a controlled orientation on the micro-hexagons like the corrugated ridges of wing scales in Morpho butterflies. Finally, the bioactivity and biocompatibility of the biomimetic hierarchical surface structures were evaluated by in vitro experiments.

## 2. Results and Discussion

### 2.1. The Characterization of Bioinspired Surface Micro/Nano-Structures

The array of micro-hexagons was directly fabricated on the Ti-6Al-4V samples by the microsecond laser to mimic the surface topology of hexagonal cells separated by deep channels in Figure 1a. Considering the previous study [28] revealing that the size of the pores on implants should be in the range of 100~200 μm for better cell adherence and osseointegration, the side length of the micro-hexagon was set to 150~300 μm and the width of channels between micro-hexagons was set to 100 μm. Furthermore, considering the corrugated ridges on the scales of Morpho butterfly wings with directional adhesion in Figure 1b, fs-LIPSS were fabricated on the array of micro-hexagons using the femtosecond laser to create nano-ripples with the controlled orientation and the spatial periodicity close to the size of cellular filopodia in the range 250~400 nm [29]. 

**Figure 2:**
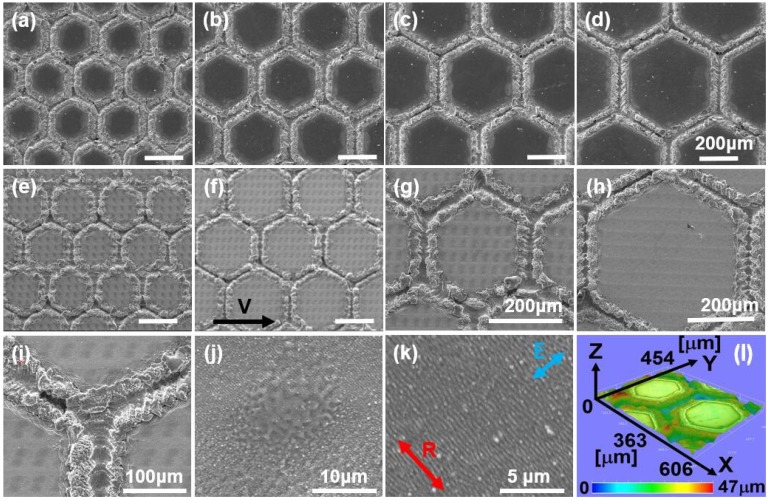
(**a**–**d**) SEM images of surface morphology of Ti-6Al-4V samples with an array of micro-hexagons at different side lengths of the hexagon: 150 μm, 200 μm, 250 μm and 300 μm, respectively, fabricated directly by the microsecond laser at the same fluence of 2000 J/cm^2^ at the scanning speed of 100 mm/min (scale bar is 200 μm in the subfigures). (**e**–**h**) SEM images of the surface morphology of the Ti-6Al-4V samples with both an array of micro-hexagons at different side lengths of the hexagon: 150 μm, 200 μm, 250 μm and 300 μm, respectively, and fs-LIPSS fabricated by the fs laser at the same fluence of 6.34 J/cm^2^ at the scanning speed of 200 mm/s and scanning intervals of 50 μm after microsecond laser processing (scale bar is 200 μm in the subfigures; the arrow V in (**f**) indicates the scanning direction of the fs laser). (**i**) SEM image of the surface morphology in the area between the micro-hexagons in the enlarged view of (**f**). (**j**) SEM image of the surface morphology of the black spot within the micro-hexagon in the enlarged view of (**f**). (**k**) SEM image of the nano-ripples in the enlarged view of (**f**) (the arrow E indicates the polarization direction of the fs laser; the arrow R indicates the orientation of the nano-ripples). (**l**) Three-dimensional morphology of (**f**).

The array of micro-hexagons at different side lengths was directly written on the Ti-6Al-4V sample surface respectively by the microsecond laser at the same fluence of 2000 J/cm^2^ in the moving speed of 100 mm/min. The samples were first analyzed by scanning electron microscopy (SEM). Figure 2a–d depict the surface micro-hexagon structures on the Ti-6Al-4V samples at different hexagon side lengths of 150 μm, 200 μm, 250 μm and 300 μm, respectively, which showed that the edges of all the micro-hexagons had greater surface roughness. To obtain the bio-inspired micro/nano-structures, fs-LIPSS were fabricated on Ti-6Al-4V samples with micro-hexagons. Figure 2e–h demonstrate the surface morphology of Ti-6Al-4V samples with micro-hexagon structures after fs laser processing at different hexagon side lengths of 150 μm, 200 μm, 250 μm and 300 μm, respectively, which showed the micro/nano-structures within the micro-hexagons. In the enlarged view of Figure 2f, the channels between the micro-hexagons had greater surface roughness in Figure 2i. Figure 2j shows the surface morphology of a black spot within the micro-hexagon in the enlarged view of Figure 2f; Figure 2k demonstrates the nano-ripples distributed within the micro-hexagons. Remarkably, the orientation of nano-ripples is perpendicular to the polarization direction of the fs laser. The three-dimensional morphology of the bioinspired surface structures in Figure 2f is depicted in Figure 2l, which shows that the average depth of channels is about 10 μm. 

**Figure 3:**
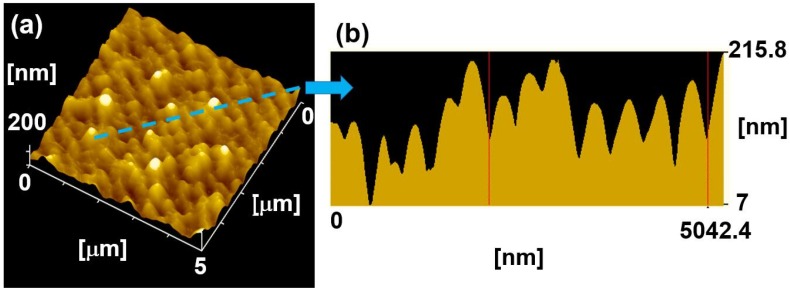
(**a**) 3D morphology of nano-ripples in Figure 2k from AFM. (**b**) The cross-sectional profile along the blue line in (**a**), indicating the depth and spatial periodicity of the nano-ripples.

The three-dimensional morphology of the nano-ripples was measured using AFM. Figure 3a shows the three-dimensional morphology of the nano-ripples in Figure 2k, which indicates that the depth of the nano-ripples is about 200 nm, and the spatial periodicity of the nano-ripples is 401 ± 20 nm, as shown in the cross-sectional profile in Figure 3b. In general, the LIPSS formation mechanism is explained by the Sipe theory representing an analytical solution for electromagnetic wave interaction with a surface scattered wave associated with the surface roughness [16,17,18], and further expanded by considering the changes of the optical properties in excited materials during ultrashort laser irradiation [13]. As a type of fs-LIPSS on Ti-6Al-4V samples, the formation mechanism of the nano-ripples has already been explained by Sipe theory with the transient dielectric permittivity of excited Ti-6Al-4V in [2].

### 2.2. In Vitro SBF Experimental Results and Analysis

**Figure 4:**
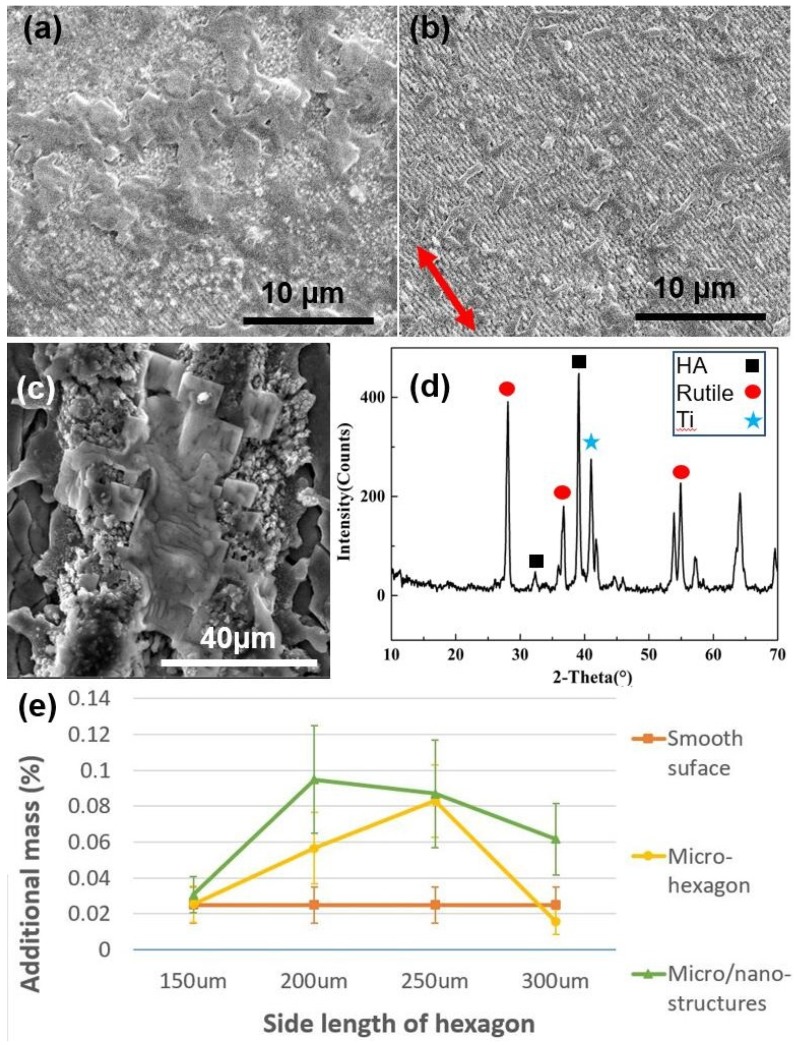
(**a**) SEM image of Ca–P surface morphology on the nano-ripples within the micro-hexagon at a hexagon side length of 200 μm after being immersed in SBF for 15 days. (**b**) SEM image of a small amount of Ca–P crystals on the nano-ripples within the micro-hexagon (the arrow indicates the orientation of nano-ripples). (**c**) SEM image of Ca–P surface morphology in the channel between micro-hexagons. (**d**) XRD spectrum of deposited Ca–P on Ti-6Al-4V samples with bioinspired micro/nano-structures. (**e**) Additional mass of deposited Ca–P on Ti-6Al-4V samples with smooth surface, micro-hexagons and micro/nano-structures at different side length of the hexagon: 150 μm, 200 μm, 250 μm and 300 μm, respectively, after being immersed in SBF for 15 days.

The in vitro experimental results indicated that the Ti-6Al-4V samples with different surface structures had different abilities to form calcium phosphate (Ca–P), which demonstrated the difference in bioactivity. After the Ti-6Al-4V samples were immersed in SBF for 15 days, Ca–P surface morphology was observed by SEM. Figure 4a shows the Ca–P surface morphology on the nano-ripples within the micro-hexagon with a hexagon side length of 200 μm. In particular, some rod-shaped Ca–P crystals were observed on the nano-ripples in Figure 4b, in which the orientation of nano-ripples was perpendicular to most of the long axes of rod-shaped Ca–P crystals. In Figure 4c, Ca–P crystals were observed on the inside surface of the channel between micro-hexagons. Furthermore, the crystal structure of deposited Ca–P was investigated by X-ray diffractometer (XRD). Figure 4d depicts the XRD spectrum of deposited Ca–P on Ti-6Al-4V samples with the composite micro/nano-structures, which shows that the deposited Ca–P was hydroxyapatite (HA). As a mineral, HA is a complex phosphate of calcium, Ca_10_(PO_4_)_6_(OH)_2_, which is the chief structural element of human and animal bones. In addition, rutile (TiO_2_) and titanium were also observed in the samples by XRD, which indicates that the oxidation process took place in the samples during laser fabrication. Finally, the increased Ca–P mass in different samples was measured by a precision balance, after being immersed in static SBF for 15 days. Figure 4e shows the comparison of the increased mass of deposited Ca–P on Ti-6Al-4V samples with different surface structures. Compared with Ti-6Al-4V samples with smooth surface and micro-hexagons respectively, the composite micro/nano-structures had more Ca–P mass. Remarkably, the micro/nano-structures with a hexagon side length of 200 μm had the maximum Ca–P mass. Apparently, the composite micro/nano-structures in SBF had a significant positive influence on bioactivity.

The best bioactivity in the composite structures can be explained by the effect of surface micro/nano-scale structures on the HA crystallization [30]. Nano-ripples in the composite structures were helpful for the aggregation of various ions in SBF to form crystal nuclei, such as PO_4_^3−^, Ca^2+^, OH^−^ and HPO_4_^2−^, then promoted the formation of HA [30]. In addition, nano-scale rutile in composite structures also facilitated the aggregation of PO_4_^3−^ and Ca^2+^ ions to form HA [31]. Therefore, the composite micro/nano-structures have an advantageous bioactivity that is useful in osseointegration.

### 2.3. Cell Culture Results and Discussion

**Figure 5:**
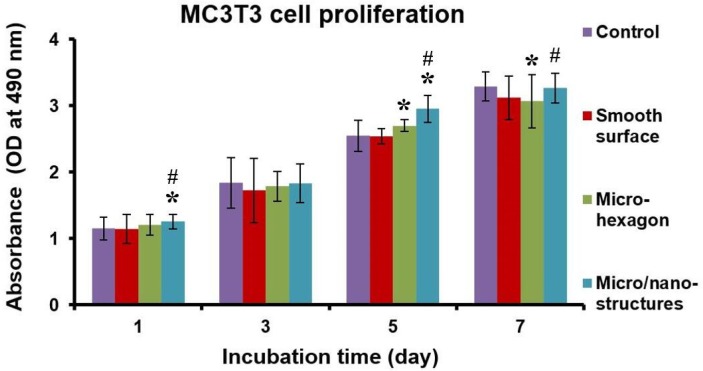
The effect of Ti-6Al-4V samples with a smooth surface, micro-hexagons and composite micro/nano-structures on cell viability evaluation using CCK-8 array (optical density, OD) at various days (1, 3, 5, 7 day) of culture. * refers to a statistically significant *p* value below 0.05 vs. control; **#** refers to a statistically significant *p* value below 0.05 vs. smooth surface.

The cell proliferation evaluation with the help of the CCK-8 array in the process of culturing on the various Ti-6Al-4V surfaces is presented in Figure 5. Experimental data characterizing cell response were the mean value of three measurements in three parallel experiments. The control group expressed the cell growing situations in the cell culture fluids without the Ti-6Al-4V sample, which was a well-grown group for the osteoblastic MC3T3-E1 cells during the whole incubation process. In Figure 5, in the whole cell proliferation process (1–7 days), the growth rate of the osteoblastic MC3T3-E1 cells on the composite micro/nano-structures was significantly higher than that on the smooth Ti-6Al-4V surface using a statistically significant difference (*p* < 0.05), which demonstrated that the biocompatibility was improved against that of the smooth surface at this stage. In detail, the optical density (OD) level of the composite micro/nano-structures at the first day of culture is higher than that on the other surfaces and the control group, which implies that the composite micro/nano-structures improved the cellular adherence. Moreover, the OD level of the composite micro/nano-structures at 5 and 7 days of culture could nearly exceed or reach that of the control group. Apparently, there was better biocompatibility on the composite structures. Therefore, the composite micro/nano-structures had a positive influence on the osteoblastic cell adherence and proliferation.

**Figure 6:**
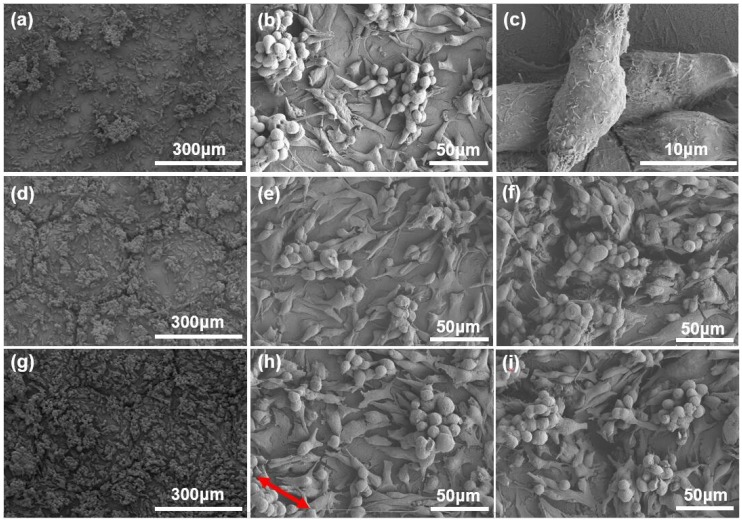
(**a**) SEM image of osteoblastic MC3T3-E1 cells grown on Ti-6Al-4V samples with smooth surface after cultivation for 7 days. (**b**) The enlarged view of (**a**). (**c**) The enlarged view of osteoblastic MC3T3-E1 cells in (**a**). (**d**) SEM image of osteoblastic MC3T3-E1 cells grown on Ti-6Al-4V samples with the array of micro-hexagons at a hexagon side length of 200 μm after cultivation for 7 days. (**e**) The enlarged view of (**d**) in the area within the micro-hexagon. (**f**) The enlarged view of (**d**) in the area near the channels between micro-hexagons. (**g**) SEM image of osteoblastic MC3T3-E1 cells grown on Ti-6Al-4V samples with composite micro/nano-structures at a hexagon side length of 200 μm after cultivation for 7 days. (**h**) The enlarged view of (**g**) in the area within the micro-hexagon (the arrow indicates the orientation of nano-ripples). (**i**) The enlarged view of (**g**) in the area near the channels between micro-hexagons.

The distribution of the grown osteoblastic cells on the specific surface topology were analyzed by SEM as follows. Figure 6 presents the SEM images of osteoblastic MC3T3-E1 cells grown on the Ti-6Al-4V samples with a smooth polished surface, the array of micro-hexagons and the composite micro/nano-structures after cultivation for 7 days, respectively. Figure 6a shows the osteoblastic MC3T3-E1 cells adherent along the surface veins and depressions on the smooth polished Ti-6Al-4V surface. In the enlarged view of Figure 6a, there are dumbbell-shaped, shuttle-shaped and spherical osteoblastic cells adherent to the Ti-6Al-4V surface. Moreover, Figure 6c shows that the shuttle-shaped cells grew on two osteoblastic cells adherent to the Ti-6Al-4V surface, which interacted with each other with many pseudopods developed beside the cell body. In comparison, Figure 6d,g show SEM images of osteoblastic MC3T3-E1 cells grown on Ti-6Al-4V samples with the array of micro-hexagons and the composite micro/nano-structures at a hexagon side length of 200 μm after cultivation for 7 days. Apparently, the density of osteoblastic MC3T3-E1 cells grown on composite micro/nano-structures in Figure 6h were higher than that of cells on the micro-hexagons in Figure 6e. In detail, in the flat area within the micro-hexagon, the density of osteoblastic cells grown on hierarchical structures in Figure 6g was much higher than that on the smooth surface in Figure 6a and the micro-hexagons in Figure 6d. This difference was attributed to the advantageous biocompatibility brought by the nano-ripples against the biological inertia of the raw Ti-6Al-4V surface. Additionally, there were many small clusters of spherical cells in the area near the channels between micro-hexagons as presented in Figure 6f,i. The channels between micro-hexagons provided space for cell growth, and the rough wall surface of channels was helpful for the cell pseudopods to adhere. Therefore, the channels of composite structures contributed to the cell adherence and growth. In regards to the above distribution of the cells and cell proliferation, the conclusion can be drawn that the composite micro/nano-structures with advantageous biocompatibility lead to the excellent adherence and growth of the osteoblastic cells on the titanium alloy.

The present study used the bioinspired composite micro/nano-structures and focused on the effect of innovative surface structures on the bioactivity, the biocompatibility and the distribution of the grown osteoblastic cells. The main differences were observed among three surface structures in the bioactivity in SBF and osteoblastic cell proliferation. Remarkably, the composite micro/nano-structures displayed the best bioactivity in SBF and excellent biocompatibility in the osteoblastic cell proliferation. From in vitro test results, it was inferred that the composite micro/nano-structures showed the greatest potential for osseointegration. The reasons for these differences between composite micro/nano-structures and the smooth surface only obtained by mechanical polishing were found in the topographical cues. At first, the channels between micro-hexagons fabricated by the microsecond laser provided the micro-scale space for cell recruitment, adhesion and orientation, and the channels of micro-hexagons increased the surface contact area, which was good for cell adherence. Secondly, after fs laser treatment, the nano-ripples formed on the surface with a spatial periodicity of 401 ± 20 nm and a depth of about 200 nm (Figure 2k, Figure 3), which was good for protein adsorption, specific integrin recruitment, the assembly of focal adhesion complexes, filopodia and lamellipodia response and further helped cell pseudopods adhere [1,2]. In addition, nano-ripples promoted the aggregation of PO_4_^3−^ and Ca^2+^ ions to form HA. Moreover, the nano-scale rutile in composite structures originating from the oxidation induced by laser fabrication also facilitated the aggregation of ions in SBF to form HA, which led to excellent bioactivity and biocompatibility. Finally, these composite architectures could stimulate osseointegration by inducing osteoblast adherence, growth and proliferation.

**Figure 7:**
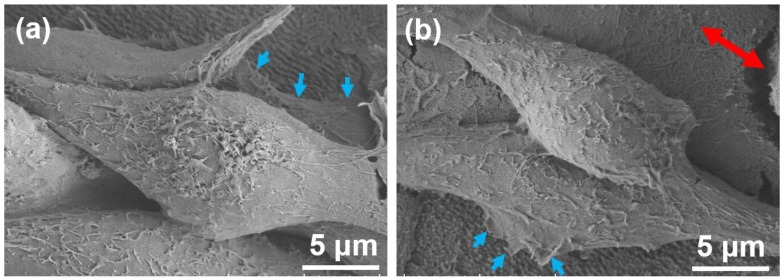
SEM images of osteoblastic MC3T3-E1 cells after cultivation for 7 days with (**a**) the focal adhesions on the nano-ripples; (**b**) the focal adhesions formed perpendicularly to the direction of the nano-ripples. (Lamellipodia and filopodia are indicated with blue arrowheads. The red arrow in (**b**) indicates the direction of the nano-ripples).

In Figure 7, cell morphology was observed by SEM in order to analyze the cell alignment. Figure 7a presents the SEM images of the bottom osteoblastic cell grown on the nano-ripples of the composite micro/nano-structures after cultivation for 7 days, in which lamellipodia and filopodia as focal adhesions interacted with the nano-ripples. Figure 7b shows that the bottom cell adherent to the substrate spread anisotropically and grew along the direction of nano-ripples, in which the focal adhesions perpendicularly to the direction of the nano-ripples were short.

Biomaterial adhesion is mediated by dynamic macro-complexes and the focal contacts, in which the initial protein interactions and the formation of focal adhesions were modulated by nanofeatures [32,33]. In this study, the mechanism of the cell alignment on the nano-ripples was explained as follows. The cell firstly probed its external environment and moved using nanoscale filopodia and lamellipodia, which were influenced by nano-topography [29]. Then, the filopodia’s ends served as anchor points for migration and the focal adhesions established. Finally, cell protrusions mostly extended in the direction of the nano-ripples, because focal adhesions that formed on the nano-ripples and extended in the direction of the nano-ripples were more stable and resulted in less filopodia retraction than when the focal adhesions were formed perpendicularly to the direction of the nano-ripples [32,33].

**Figure 8:**
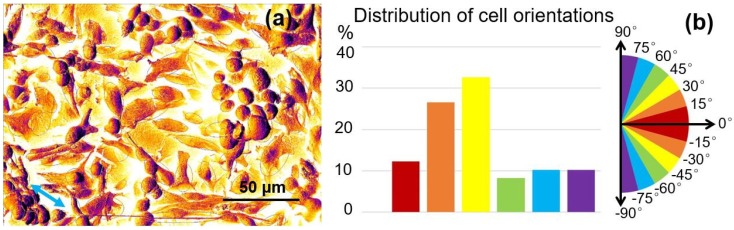
(**a**) The processed image of osteoblastic cells grown on the nano-ripples within the micro-hexagon, with the additional fitted ellipses by ImageJ software based on Figure 6h (the arrow indicates the orientation of the nano-ripples). (**b**) The distribution of cell orientations. The orientation angle is obtained from the angle between the direction of the nano-ripples, predefined as 0°, and the major axis of the cell measured with ImageJ software.

Concerning the cell alignment on the nano-ripples after cultivation for 7 days, the distribution of osteoblastic cell orientations in Figure 6h were processed and analyzed using ImageJ software. To obtain the cellular orientation, the cells with a cell surface area *A* > 100 μm^2^ and shape factor *S* of 0–0.7 were identified and fitted with ellipses by ImageJ, as shown in Figure 8a. Figure 8b presents the statistical distribution of cell orientations in Figure 8a, which indicated that 77.6% of the cells were oriented at −45°~+45° from the direction of the nano-ripples predefined as 0°. Therefore, it can be concluded that the nano-ripples were able to induce cellular alignment.

However, in Figure 8b only a small number of cells were oriented at 0°, whereas most cells were oriented at −45°~+45°. This is because the shape of the nano-ripple could perturb the cell alignment by perturbing the direction of the filopodia [34]. Considering the imperfect shape of self-organized nano-ripples in Figure 3, there was a diverging distribution of cell orientations in relation to the nano-ripples.

Numerous previous studies have already demonstrated the ability of cells to align with nano-grooves, known as contact guidance [35]. Cell alignment was correlated with the alignment of focal adhesions and actin filaments along the substrate nano-topographies, which provided more opportunities for focal attachment. As a mechanical constraint, the nano-topography greatly changed the cellular morphology, cytoskeleton dynamics and even the signals that controlled the cell adhesion, growth and differentiation [36]. In this work, the anisotropic distribution of osteoblastic cell orientations could be an advantage for different biological applications, for example, a higher mechanical strength along the direction of nano-ripples on the implant surface. The mechanical properties play a key role in the long-term performance of the implants [37]. Because the depth of composite micro/nano-structures is less than 20 μm, the entire mechanical properties of Ti-6Al-4V samples changed a little. In addition, the laser surface processing could induce the residual stresses that may improve fatigue strength [38].

Bioinspired by the skin structures in tree frog toe pads with excellent isotropic adhesion and the corrugated ridges on the scales of Morpho butterfly wing with anisotropic adhesion, the strategy of controlling the osteointegration on the titanium alloy implants was proposed using the composite micro/nano-structures as follows. The bioinspired composite micro/nano-structures on titanium alloy consisted of the array of micro-hexagons and the nanoripples with the desired orientation and the spatial periodicity of 401 ± 20 nm, which were respectively fabricated by microsecond and femtosecond lasers. The micro-hexagon channels provided the microscale space for cell recruitment, adhesion and proliferation, which enhanced the mechanical interlocking of the bone–implant interface and promoted the subsequent cell growth within hexagonal areas. Moreover, osteoblastic cell adherence and proliferation were again improved by the nano-ripples with the nanoscale rutile induced by laser processing. Remarkably, the nano-ripples were able to induce cellular alignment within the micro-hexagons, giving the advantage of better mechanical strength along the direction of nano-ripples on the implant surface. Therefore, both the array of micro-hexagons and nano-ripples in the composite micro/nano-structures have a positive influence on the bioactivity and biocompatibility of Ti-6Al-4V implants, which helps with controlling the osteointegration. Moreover, this functionalization strategy could be meaningful in a range of regenerative medicine and tissue engineering applications.

## 3. Experimental

### 3.1. Materials

The surface-polished commercial titanium alloy Ti-6Al-4V sheets (ASTM Grade 5 without ELI, Baoji Titanium Industry Co. Ltd., Baoji, China) with the size of 20 mm × 20 mm × 1 mm were used in this work. Before and after laser irradiation, the samples were cleaned ultrasonically for at least 15 min with acetone, ethanol and deionized water successively. Finally, the samples were dried in air. 

### 3.2. Laser Surface Structuring

Biomimetic surface microstructures on the Ti-6Al-4V samples were first fabricated by a microsecond laser using a SPI fiber laser system (SPI-100C) at a wavelength of 1070 nm with a pulse width of 500 μs. The sample was irradiated by the laser beam normal to the surface, with a focus spot of about 18 μm diameter. The laser fluence of 2000 J/cm^2^, the scanning speed of 100 mm/min and repetition rate of 1000 Hz were used in the experiments.

Fs-LIPSS were fabricated using a femtosecond laser (Light Conversion, PHAROS, PH1-10) with a wavelength of 1030 nm, a pulse width of 220 fs and a repetition rate of 100 kHz. The linearly polarized laser beam was focused by a F-Theta lens of focus length F = 100 mm to a spot size about 25 μm. The laser fluence was adjusted by using the combination of a waveplate and a polarizer. A scanning galvanometer was used to control the Gaussian laser beam scanning with a scanning rate of 200 mm/s and scanning intervals of 50 μm. The laser processing at laser peak fluence of 6.34 J/cm^2^ was carried out in air at ambient temperature and pressure to create nano-ripples with a controlled orientation on the Ti-6Al-4V samples, which had been processed by the microsecond laser.

### 3.3. Surface Morphology and Crystal Analysis

A scanning electron microscope (SEM, FEI Q45, Hillsboro, OR, USA) and atomic force microscope (AFM, SPI3800N, Seiko Instruments, Inc., Chiba, Japan) were used to study the surface morphology of the samples. SEM was operated at 15 kV under high vacuum. A three-dimensional surface region of 5 μm × 5 μm was acquired by AFM in contact mode using standard silicon tips. The crystal structures of samples were investigated by X-ray diffractometer (XRD, Japan Rigaku Corporation, D-max-2200PC, Tokyo, Japan) with CuKα radiation of wavelength 1.54 Angstrom in out of plane geometry over the 2θ range of 10°–70° at a scanning speed of 8°/min.

### 3.4. In Vitro SBF Experiments

Simulated body fluid (SBF) was prepared in the experiments following a conventional recipe by Kokubo et al. [39,40] as follows. Chemicals were dissolved in one liter of distilled water in the sequence of NaCl (8.035 g), NaHCO_3_ (0.355 g), KCl (0.225 g), K_2_HPO_4_∙3H_2_O (0.231 g), MgCl_2_∙H_2_O (0.311 g), TRIS (tris-hydroxymethyl aminomethan) (6.118 g), CaCl_2_ (0.292 g), Na_2_SO_4_ (0.072 g) and 1 mol/L HCL. The fluid was buffered at pH 7.40, 36.5 °C, with TRIS and 1 mol/L HCL. The ability to form calcium phosphate (Ca–P) was evaluated in static SBF environments. The SBF in the storage tank was replaced with fresh SBF every two days in order to keep the solution ion concentration stable. After Ti-6Al-4V samples with different surface treatment were immersed in static SBF for 15 days, the increased Ca–P mass on different samples was measured by a precision balance.

### 3.5. In Vitro Cell Culture Tests

MC3T3-E1 cells (Yeasen biotech Co. Ltd., Shanghai, China) were purchased and used for cell adherence, orientation and proliferation studies. MC3T3-E1 cells are osteoblastic cells from mouse calvaria, which typically retain strong similarities with primary cells and are thus an established in vitro model of osteoblasts. Blank culture medium (as control) and Ti-6Al-4V samples with different surface treatment were used for the following in vitro rat cell culture tests. Before the tests, all samples were sterilized by soaking in 75% ethanol for 60 min, followed by 30 min UV exposure. After continuous cell culture of 48 h, the cell suspension of MC3T3 was prepared with the digestion of 0.25% trypsin. The manipulated cell density of 5 × 10^4^/mL was inoculated on each hole of the culturing plate. Then, the transferred cells were pre-cultured in the cell culturing box in an environment of 37 °C and 5% CO_2_, which was prepared to make the cell adherence to the vessel wall for the subsequent experiments.

After rinsing twice in sterile ultrapure water, different samples were placed in the 48-well plates and seeded with cell suspension containing about 5 × 10^4^ cells. The culture medium was also changed every day. MC3T3 cells were cultured for 1, 3, 5 and 7 days, in order to determine the proliferation rate. After 1, 3, 5 and 7 days of culture, the cells on the material surface were polished with sterile PBS (phosphate buffer solution) to the WST-8 solution for cultivation in the 37 °C environment for 2 h. The cell count kit-8 (CCK-8), a simplified assay using tetrazolium salt, was employed for quantitative evaluation of cell viability as the proliferation behavior on various samples after incubation. WST-8 solution was added to each well and incubated at 37 °C for 6 h, and then absorbance of the formed formazan at 490 nm was monitored with the help of a microplate reader. The control group was set in parallel without the titanium alloy sample in the cell culture.

After 7 days of culture, the cell-seeded samples were removed from the medium and gently rinsed with PBS. The cells cultured on the samples were then fixed with 2.6% PBS solution of glutaraldehyde at 4 °C for 2 h. After the fixative was removed, the samples were subsequently rinsed gently with PBS and ultrapure water. Then, dehydration for 10 min with ethanol in a series of gradient concentration solutions was performed. The samples were then dried for 2 days and coated with gold for 60 s prior to SEM observation at an accelerating voltage of 3 kV. The surface topographic effect of samples on cell adhesion, orientation and proliferation was investigated by SEM (Hitachi SU8010). The following reagents were used: DMEM culture medium (Gibco, Thermo fisher scientific Inc., Waltham, MA, USA); trypsin (Amresco Inc., Solon, OH, USA, 0.25%); CCK-8 kit (Yeasen biotech Co. Ltd., Shanghai, China); standard FBS (HyClone, Logan, UT, USA, 10%); PBS (Wako pure chemical industries, Ltd., Osaka, Japan); ultrapure water (Self-made, Heal force bio-meditech holdings Ltd., Shanghai, China); ethanol (Analytically pure, Fuyu chemical Ltd., Dongying, China); NaHCO_3_ (Analytically pure, Sigma-aldrich LLC., Munich, Germany); acetone (Analytically pure, Baiyin liangyou chemical reagents Co. Ltd., Baiyin, China) and H_2_SO_4_ (Baiyin liangyou chemical reagents Co. Ltd., Baiyin, China, 98%).

### 3.6. Image Analysis

Morphometric measurements including the cell orientation and cell number were performed with the ImageJ software to obtain quantitative information [41]. For cell numeration, a threshold was applied to the SEM image to finish image binarization and the particle analyzer in ImageJ software was used to identify cells and the fitted ellipses based on cellular shape and area in each image. In detail, morphometric parameters such as the cell surface area *A* and cell perimeter *P*, were automatically measured by ImageJ program after image binarization, and shape factors were generated from these data. The shape factor *S = 4 π (A/P^2^)* assesses the deviation from circularity: a circle has a value of *S = 1*, and *S* approaching 0.0 indicates an increasingly elongated polygon. To obtain the cellular orientation, the longest axis for each cell with the fitted ellipse was automatically defined so that the program could calculate the angle made by its major axis. Data transformation using Microsoft Excel rendered it possible to obtain the angle of this cellular axis according to a predefined 0°-axis corresponding to the direction of the nano-ripples [41].

### 3.7. Statistical Analysis

Data from experiments characterizing the cell response are presented as the mean ± standard error (SE) of all the measurements performed for three independent cultures on different samples. All experiments were repeated at least three times to ensure validity of the observations. Data were evaluated by analysis of variance, and significant differences between groups were determined using Student’s *t*-test. *p* < 0.05 was considered to indicate a statistically significant difference.

## 4. Conclusions

Inspired by both the adhesive skin structures of tree frog toe pads and the corrugated ridges on the scales of Morpho butterfly wings with anisotropic adhesion, composite micro/nano-structures, including the array of micro-hexagons and oriented nano-ripples on Ti-6Al-4V samples, were fabricated by microsecond laser direct writing and fs-LIPSS, which resulted in both good bioactivity in simulated body fluids and excellent biocompatibility in cell proliferation. An innovative functionalization strategy of controlling the osteointegration on titanium alloy implants was proposed using the composite micro/nano-structures, in which the channels of micro-hexagons provided the microscale space for cell recruitment, adhesion and proliferation, enhancing the mechanical interlocking of the bone–implant interface. Furthermore, the nano-ripples were able to induce cellular alignment within the micro-hexagons for better directional mechanical strength on the implant surface. Thus, this innovative method enabled the creation of controlled, reproducible, bioinspired micro/nano-topographies to improve the adhesion, alignment and proliferation of osteoblastic cells on Ti-6Al-4V implants, without chemically altering the titanium alloy, which is supposed to be meaningful in a range of regenerative medicine and tissue engineering applications, including orthopedics and dentistry.
